# Injectable Poloxamer Hydrogels for Local Cancer Therapy

**DOI:** 10.3390/gels9070593

**Published:** 2023-07-24

**Authors:** Ana Camila Marques, Paulo Cardoso Costa, Sérgia Velho, Maria Helena Amaral

**Affiliations:** 1 UCIBIO—Applied Molecular Biosciences Unit, MEDTECH, Laboratory of Pharmaceutical Technology, Department of Drug Sciences, Faculty of Pharmacy, University of Porto, R. Jorge Viterbo Ferreira 228, 4050-313 Porto, Portugal; 2 Associate Laboratory i4HB—Institute for Health and Bioeconomy, Faculty of Pharmacy, University of Porto, R. Jorge Viterbo Ferreira 228, 4050-313 Porto, Portugal; 3 i3S—Instituto de Investigação e Inovação em Saúde, University of Porto, R. Alfredo Allen 208, 4200-135 Porto, Portugal; 4 IPATIMUP—Institute of Molecular Pathology and Immunology of the University of Porto, R. Júlio Amaral de Carvalho 45, 4200-135 Porto, Portugal

**Keywords:** cancer therapy, injectable hydrogels, poloxamer, intratumoral administration

## Abstract

The widespread push to invest in local cancer therapies comes from the need to overcome the limitations of systemic treatment options. In contrast to intravenous administration, local treatments using intratumoral or peritumoral injections are independent of tumor vasculature and allow high concentrations of therapeutic agents to reach the tumor site with minimal systemic toxicity. Injectable biodegradable hydrogels offer a clear advantage over other delivery systems because the former requires no surgical procedures and promotes drug retention at the tumor site. More precisely, in situ gelling systems based on poloxamers have garnered considerable attention due to their thermoresponsive behavior, biocompatibility, ease of preparation, and possible incorporation of different anticancer agents. Therefore, this review focuses on the use of injectable thermoresponsive hydrogels based on poloxamers and their physicochemical and biological characterization. It also includes a summary of these hydrogel applications in local cancer therapies using chemotherapy, phototherapy, immunotherapy, and gene therapy.

## 1. Introduction

Local cancer therapy holds great potential to address the shortcomings of systemic treatment options, namely the lack of specificity for the target, low therapeutic efficiency, and drug resistance.

Different from intravenous (IV) administration, local treatments using intratumoral (IT) or peritumoral (PT) injections allow high concentrations of therapeutic agents to reach the tumor site, bypassing the bloodstream and non-specific interactions with healthy tissues [1]. Moreover, because local therapies are independent of tumor vasculature, delivery is not restricted to tumor regions with better perfusion. Besides increasing the stability of anticancer agents, local administration also allows for the use of novel combinations of co-solvents and polymers for solubilization, encapsulation, and incorporation of water-insoluble drugs.

In contrast to delivery systems based on implants (wafers, rods, and films) and particles, injectable biodegradable hydrogels require non-surgical procedures and promote retention of free or encapsulated drugs at the tumor site [2]. Indeed, treatment with injectable cisplatin (CDDP)/epinephrine gel has been proven practicable by direct injection into superficially accessible tumors or endoscopically for esophageal cancer [3]. Regarding the distribution dynamics, nanoparticles embedded in hydrogels were observed to cover larger areas of the tumor than free nanoparticles upon IT administration [4].

Injectable gels include pre-formed gels with shear-thinning and self-healing properties [5] and in situ-forming gels [6]. After injection as free-flowing polymer solutions, in situ gelling systems transform into gels at the injection site, acting as drug depots for sustained drug release. Generally, the mechanism of depot formation is an in situ phase transition triggered by external stimuli such as changes in temperature.

In this review, we emphasize the use of poloxamers in the development of injectable thermoresponsive hydrogels and their characterization. The extensive application of injectable poloxamer hydrogels in local cancer therapy, including chemotherapy, phototherapy, immunotherapy, and gene therapy, is also summarized.

## 2. Poloxamer-Based Thermoresponsive Hydrogels

Hydrogels are thermoresponsive if they respond to changes in temperature. A case in point is thermoresponsive polymer solutions exhibiting a sol–gel phase transition upon the shift from room to body temperature (37 °C). The temperature at which the transition from one phase to two phases (polymer-enriched and aqueous phases) takes place is termed the “critical solution temperature” of the polymer [7].

Not many thermoresponsive polymers show an upper critical solution temperature (UCST) in aqueous media under relevant conditions. Conversely, most thermoresponsive polymers having a phase transition temperature near 37 °C present a lower critical solution temperature (LCST) in water. When raising the temperature above the LCST, these polymers become insoluble and separate from the solution, yielding a hydrogel. During this process, polymer–solvent interactions based on hydrogen bonds are weakened, which leads to partial dehydration and aggregation of polymer chains. The progressive exclusion of water molecules with heating will expose the hydrophobic polymer domains and thus facilitate the establishment of polymer–polymer hydrophobic interactions (hydrophobic effect). Gelation is reversible at temperatures below the LCST because polymers become miscible with water again [8,9].

The basic requirements of in situ gelling systems for local administration are injectability and gelation under physiological conditions. Therefore, in addition to allowing the incorporation of therapeutic agents, thermoresponsive polymer solutions should have low viscosity to flow easily during administration and rapidly form a gel once injected [1]. Although other in situ-forming thermoresponsive hydrogels based on chitosan [10,11], polyacrylamides [12], and polyesters [13,14] have been developed for this purpose, poloxamers have gained significant attention from researchers over the past two decades.

**Figure 1:**
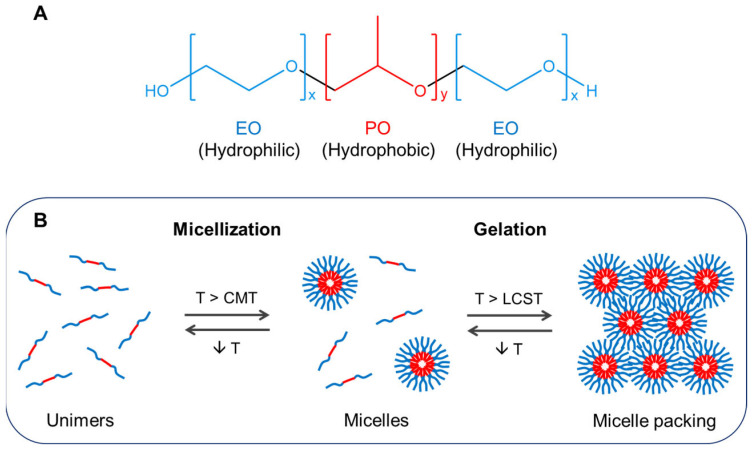
Schematic of the structure and reversible thermoresponsive behavior of poloxamers. (**A**) Poloxamers comprise a central block of “y” numbers of propylene oxide (PO) units and two side blocks of “x” numbers of ethylene oxide (EO) units. (**B**) Heating above the critical micellization temperature (CMT) and above the lower critical solution temperature (LCST) of poloxamer induces micellization and gel formation by micelle packing, respectively. Reprinted from [17], Copyright 2019, with permission from Elsevier.

This family of poly(ethylene oxide)-poly(propylene oxide)-poly(ethylene oxide) (PEO PPO-PEO) triblock copolymers (Figure 1A) is synthesized by sequential ring-opening polymerization of propylene oxide and ethylene oxide monomers, using an alkaline catalyst (i.e., potassium or sodium hydroxide) [15]. A series of poloxamers diverging in molecular weight and PPO/PEO ratio is available with different trade names like Pluronic, Lutrol, or Synperonic [16].

The individual PEO-PPO-PEO block copolymers (unimers) self-assemble into micelles above a “critical micelle concentration” because of their amphiphilic character in aqueous solutions. Structurally, the micellar core comprising hydrophobic PPO blocks is surrounded by a hydrophilic PEO shell that forms hydrogen bonds with the water molecules. Due to the nanosized and core-shell structure of poloxamer micelles, it is feasible to entrap hydrophobic drugs in the core and carry them into an aqueous environment [15]. Increasing temperature to the “critical micellization temperature” also induces micelle formation by favoring the dehydration of PPO groups and their interaction via van der Waals forces [17]. At a fixed PPO/PEO ratio, the longer the PPO block, the lower the temperature and the poloxamer concentration needed to form micelles in water [18]. The effect of temperature on micellization is reversible because cooling causes an equilibrium shift from micelles back to unimers. Upon further heating above the LCST, micelles aggregate and entangle to become a gel (Figure 1B).

The rich phase behavior of poloxamer dispersions makes them a versatile platform for drug delivery in the form of micelles or hydrogels, which have enduring popularity in cancer therapy [19,20,21]. However, before considering the development of pure poloxamer hydrogels, researchers should be aware of their limitations under high compression or in aqueous media due to low mechanical strength and fast erosion, respectively [22]. Notwithstanding that thermosensitivity is appealing in the biomedical field, this property depends on concentrated aqueous dispersions of poloxamers [15]. Moreover, to impart biodegradability to the hydrogel, modifications in the polymer structure can be considered [23].

Among poloxamers, poloxamer 407 (P407) or Pluronic^®^ F127 (PF127) solutions are the most formulated for IT or PT injection on account of their reversible thermoresponsive nature that allows gelation close to the physiological temperature (at concentrations of 20% (*w*/*w*) or less) and residence at the site of implantation as a sustainable carrier [18,24]. Accordingly, P407 exists in a sol state under colder conditions, during which it can be loaded with therapeutic agents for posterior release from its gel form. Moreover, the matrix structure of P407—and thus, the release kinetics of therapeutics—can be modified with other polymers by altering the hydrogel degradation rate [25]. P407, with a molecular weight of ~12.6 kDa, has been approved as an excipient by the U.S. Food and Drug Administration (FDA) for pharmaceutical applications and is listed in the US and European Pharmacopoeia [15,26]. The molecular weight and end group identity of P407 can also be tailored to adjust gelling and adhesiveness properties [27].

## 3. Characterization of Injectable Poloxamer Hydrogels

Typically, poloxamer hydrogels are prepared according to the “cold method”, consisting of adding an appropriate amount of polymer to water or phosphate-buffered saline (PBS) solution for blank hydrogels, or to nanoparticle dispersions for nanocomposite hydrogels, under stirring at 4 °C until a clear dispersion is obtained [28,29]. It is also possible to mix free drugs or drug-loaded nanoparticles with preformed polymer dispersions cooled to 4 °C. Then, the obtained poloxamer dispersions are usually characterized regarding sol–gel transition, rheological properties, in vitro degradation, and release profile.

### 3.1. Gelation Temperature

Different techniques have been used for measuring the sol–gel transition temperature (T_sol-gel_). The simplest is the test tube inversion method, where each formulation is sealed in a test tube and heated slowly in a water bath [30]. The temperature at which no flow is observed as inverting the tube is considered the gelation temperature. Alternatively, poloxamer formulations are gradually heated under stirring in a beaker containing a small magnetic bar. Once the magnetic bar stops moving, the recorded temperature is referred to as T_sol-gel_ [31]. Inasmuch as observation of gelation is straightforward and requires minimal equipment, visual methods lack precision and accuracy [17]. More reliable methods for investigating phase transition include rheological and differential scanning calorimetry (DSC) measurements. Although temperature-controlled rotary viscometers can be used [32], T_sol-gel_ is determined more precisely by oscillatory shear rheology, measuring the elastic (or storage) modulus (G′) and viscous (or loss) modulus (G″) of the hydrogel. In oscillation mode, gelation can be detected as a function of temperature or time, with the G′/G″ crossover point indicating the gelation point. Contrastingly, the gelation process is evidenced by a secondary endothermic peak in the DSC thermogram with no insight into changes in the rheological behavior [17]. To be suitable for in vivo applications, gelation temperature should be near body temperature but never above 37 °C. Otherwise, the sol–gel transition might not occur at the injection site, resulting in leakage of the poloxamer formulation to the surrounding tissues. The T_sol-gel_ values are also expected to be higher than room temperature to avoid premature gelation that impedes injection [31]. It is established that poloxamer concentration and gelation temperature vary inversely [33]. Therefore, the combination of PF407 with poloxamer 188 (Pluronic^®^ F68, PF68) is often recommended to obtain an acceptable gelation temperature, which also increases gel strength versus the polymers used alone [34].

### 3.2. Rheological Behavior, Mechanical Strength, and Injectability

As expected, the rheological properties of poloxamer dispersions are temperature-dependent, exhibiting Newtonian behavior at low temperature and non-Newtonian, shear-thinning behavior at higher temperatures [31,35]. The mechanical strength of the hydrogels is essential for maintaining their integrity in the body, but is usually overlooked during characterization. Nevertheless, in vitro degradation and release studies provide evidence of low gel strength. As a result of rapid erosion, there is a burst release behavior, meaning the fast release of a considerable fraction of payload into a hydrolytic medium that simulates human physiological conditions (PBS at 37 °C) [36,37]. Of note, as to delivery to tumor cells, the tumor microenvironment is more closely imitated if acidity (pH 6.8) and the presence of enzyme hyaluronidase are considered [38]. Some researchers sought to improve the mechanical properties of poloxamer hydrogels by introducing a chain extender (hexamethylene diisocyanate, abbreviated to HDI) into the polymer [39] or adding bioadhesive polymers such as N,N,N-trimethyl chitosan [40], alginate [41], or xanthan gum [38]. Ju et al. [42] upgraded this strategy and prepared a P407 hydrogel interpenetrated by a network of carboxymethyl chitosan crosslinked with glutaraldehyde but losing thermosensitivity. Instead, chitosan can be crosslinked with genipin to form an interpenetrating scaffold within P407 hydrogels [43].

Notwithstanding that injectability is a critical parameter of the injection performance, not many authors assess the force required to perform the administration via a syringe [44]. Those who do either evaluate the easiness of passing the hydrogel through a needle in qualitative terms [45] or conduct uniaxial tensile testing using a mechanical testing machine with a syringe fitted with a needle or catheter [46].

### 3.3. Biocompatibility and Sterilization

The safety and non-toxicity of poloxamer hydrogels for local tumor administration have been observed in vitro [47,48] and in vivo through histological examination for signs of inflammation after subcutaneous implantation [39,49]. In addition to biocompatibility, sterility is a requirement for considering the potential clinical use of any material intended to be in close contact with the human body [50]. However, the impact of sterilization on poloxamer hydrogels has been understudied. To date, steam sterilization (121 °C, 15–20 min) was the most investigated, which was found to cause a slight decrease in gelation temperature [25,51]. This observation can be explained by an increase in polymer weight fraction due to water evaporation during autoclaving. It was suggested that autoclaving at a lower temperature for a longer time (e.g., 105 °C for 30 min) would allow the poloxamer hydrogels to be more like the non-sterilized ones [52].

## 4. Local Tumor Administration of Poloxamer Hydrogels

The proof-of-concept of poloxamer hydrogels for local tumor administration has been demonstrated by the growth inhibition of several tumors in different mouse models. Nevertheless, it is noteworthy that most tumor models were established in mice through the subcutaneous inoculation of cancer cells. Subcutaneous (or ectopic) tumors might be advantageous to monitoring tumor growth and performing local injections, but fail to mimic the tumor microenvironment. Despite being more clinically relevant because tumor xenografts are placed in the tissue/organ of origin, orthotopic mouse models still do not reflect the size of tumors that develop naturally in patients. Moreover, potential adverse effects in cancer patients with intact immunity may go unnoticed if studies in immunodeficient animals are the case [53].

**Figure 2:**
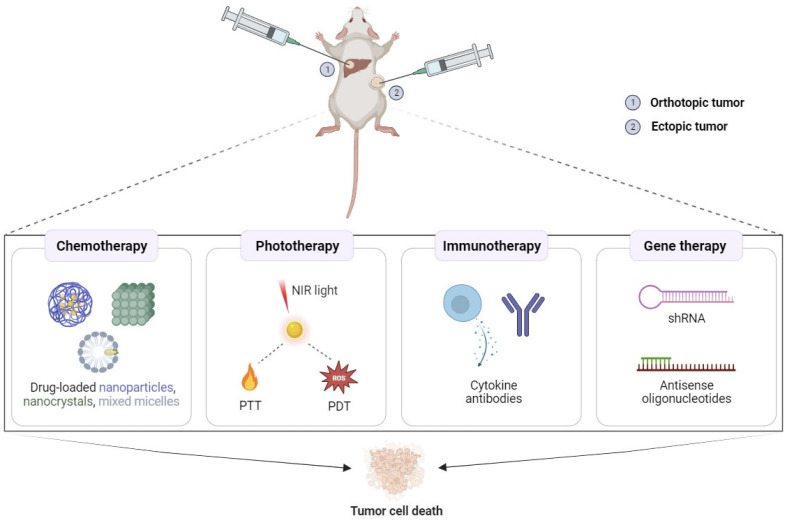
The in vivo administration of poloxamer hydrogels for local cancer therapies including chemotherapy, photothermal therapy (PTT), photodynamic therapy (PDT), immunotherapy, and gene therapy.

The application of injectable poloxamer hydrogels in local cancer therapy is depicted in Figure 2 and discussed below, with several examples organized by therapeutic modality.

### 4.1. Potential Applications in Cancer Chemotherapy

For local cancer chemotherapy, P407 or PF127 solutions were mixed with free drugs, such as paclitaxel (PTX) [32], topotecan [54], doxorubicin (DOX) [55], and salinomycin [47]. Still, most PF127 hydrogels reported for IT or PT injection accommodate anticancer drugs encapsulated in nanoparticles [56,57], nanocrystals [28,58,59], cyclodextrin inclusion complexes [60,61], hyaluronic acid-based nanocomplexes [62,63], and mixed micelles [63,64]. The literature also contains several examples of in situ-forming gels using mixtures of PF127 and PF68 for the local delivery of free [45,65] and encapsulated [30,31,49,66] drugs.

The direct incorporation of free PTX into a P407 solution at the final concentration of 0.5 mg/mL, albeit simple, resulted in a very slow in vitro drug release from P407 hydrogel because of the poor water solubility of PTX. Moreover, although it was completely dissolved at lower concentrations, PTX formed a suspension when the final concentration was doubled (1.0 mg/mL) [32]. This reflects the low solubilization of hydrophobic drugs in P407, which generally imparts limited drug loading and physical instability to poloxamer micelles. Therefore, hybrid systems integrating drug-loaded nanoparticles and thermoresponsive hydrogels have been intensively studied to improve drug release and increase drug loading capacity [28,67].

The combination of liposomes and poloxamer hydrogels was proposed to stabilize the lactone form of 7-ethyl-10-hydroxycamptothecin [66] and prolong the release of PTX [49,56]. Whereas PF127/PF68 hydrogels enhanced the retention of drug-loaded liposomes at the tumor site [49,66], the use of liposomes made of 0.21–1.25% soybean phospholipids was suggested to allow a 3–9 wt% decrease in the poloxamer concentration required for an in situ-forming PF127 gel [56]. Notwithstanding the evidence from studies in MCF-7 breast cancer cells supporting the higher cytotoxic activity of tamoxifen citrate-loaded niosomes compared to the free drug, the low viscosity of niosomal suspensions prompted their dispersion into poloxamer hydrogels [31]. In a very interesting approach to the treatment of melanoma, Yu et al. [57] prepared a PF127 hydrogel to intratumorally deliver CDDP-loaded poly(α-L-glutamate)-g-mPEG nanoparticles and microspheres entrapping losartan potassium that exerts antifibrotic effects, namely by inhibiting the production of collagen I in tumors. The incorporation of both microspheres and nanoparticles into the gel enabled most losartan to be released first and reduce the collagen content prior to the release of CDDP, which occurred in the following days after the nanoparticles have penetrated more deeply into the tumor. Differently, Shen et al. [68] combined nanotechnology and active targeting with thermoresponsive polymers for IT administration of PTX in pancreatic tumors. For that, they prepared a PF127/PF68/hydroxypropyl methylcellulose gel bearing PTX-loaded mPEG-poly(D,L-lactide-co-glycolide)-poly(L-lysine) nanoparticles functionalized with a cyclic peptide, which specifically binds to αvβ3 integrin overexpressed on the endothelial tumor cells. Later, Xie et al. [69] also developed a PF127/PF68/hydroxypropyl methylcellulose hydrogel to improve the efficacy and safety of norcantharidin (NCTD) for treating hepatic cancer. In another work, Gao et al. [29] took into consideration that NCTD has poor solubility in water, thereby preparing NCTD-loaded polymeric nanoparticles before incorporating them into a DOX-containing PF127 hydrogel to treat hepatocellular carcinoma via IT administration.

The formulation and dispersion of drug nanocrystals into PF127 hydrogels deserved some attention, considering that nanocrystals provide higher drug loading than other nanocarriers [28,58]. Further, Gao et al. [58] dissolved D-α-tocopherol PEG 1000 succinate in PF127 solutions to impair drug efflux and reverse drug resistance of P-glycoprotein-overexpressing liver cancer cells. Together with lapatinib-loaded microparticles, PTX nanocrystals were incorporated into PF127 hydrogel for PT injection to imitate the slow and fast release of these two drugs in clinical use [59].

Attempts to increase the water solubility of the anticancer agent β-lapachone involve the formation of inclusion complexes with cyclodextrins. Intending to design injectable thermoresponsive hydrogels containing β-lapachone, Landin’s group used Artificial Neural Network modeling to understand the interactions between the polymer (PF127) and the solubilizing agent (cyclodextrin) and obtain the optimal formulation [60]. A significant decrease in cell viability and tumor volume was observed following the treatment of MCF-7 cells and in the breast xenograft mouse model with this ternary system [61]. When studying the effect of methylated β-cyclodextrin and ethanol on the β-lapachone solubility and gel properties, these authors confirmed that both additives promote drug solubilization [70]. However, the addition of ethanol as a co-solvent may render Pluronic^®^ (F127 and P123) dispersions inappropriate for IT administration. Data from rheological characterization suggested that autoclaving may not affect the gelation temperature and gel strength of Pluronic^®^ systems with β-lapachone [70].

An injectable PF127 hydrogel containing DOX complexed with HA and MgCl_2_ was developed by Jhan et al. [62] and was demonstrated to cause the growth inhibition of C26 colon cancer cells in a mouse model. This drug delivery system was patented (US9364545B2) [71] and then ameliorated by adding a mixed micellar formulation composed of PF127 and Pluronic^®^ L121 for carrying a second chemotherapeutic drug (DTX) [63]. Mixed micelles consisting of PF127 and another surfactant, such as Solutol^®^ HS15 [30], Tween^®^ 80 [64], or D-α-tocopherol PEG 1000 succinate [72], have been incorporated into PF127 hydrogels to deliver hydrophobic drugs, namely DTX [30,64] and PTX [72].

By synthesizing the dalteparin-P407 copolymer, Li et al. [73] repositioned low-molecular-weight heparin as an anticancer agent and fabricated a novel thermosensitive and injectable hydrogel carrying DOX-loaded laponite nanoparticles.

Only one of the articles reviewed [74] reported the use of poloxamer hydrogels for local chemoradiotherapy. The concurrent IT administration of chemotherapeutics and radiation was achieved by using PF127 hydrogels co-loaded with DOX and gold nanoparticles.

### 4.2. Potential Applications in Cancer Phototherapy

Considering the mechanisms of light conversion, phototherapy includes photothermal therapy (PTT) and photodynamic therapy (PDT). Phototherapy based on PTT or PDT can eliminate cancer cells by generating hyperthermia or reactive species of oxygen (ROS) [75,76].

PTT involves the laser activation of photothermal agents, followed by near-infrared (NIR) light conversion into heat. Despite great progress in cancer PTT, most photothermal agents are made of heavy metals and given intravenously, causing safety concerns to arise. To reduce putative systemic toxicity and enhance local retention, Fu and colleagues indicated PF127 hydrogels embedding copper sulfide nanodots [77] or Prussian blue nanospheres [78] for PT administration. Given that seaweed polysaccharides have good biocompatibility, biodegradability, and non-toxicity, Chen et al. [79] prepared an injectable photothermal hydrogel using iota carrageenan-capped gold-silver nanoparticles and PF127. The in vivo results pointed to a multifunctional hydrogel that could prevent tumor growth and recurrence and promote post-surgical wound healing without chemotherapeutic drugs and antibiotics [79]. An alternative to metal nanoparticles as photothermal agents is organic agents (i.e., indocyanine green), but their intrinsic instability limits their therapeutic effects. As a result, organic–inorganic hybrid nanomaterials such as titanium carbide (Ti_3_C_2_) nanoparticles have received attention and have been combined with PF127 through a simple mixture to form an injectable hydrogel for local PTT [80].

The combination of chemotherapy with other therapeutic modalities, namely phototherapy, has gained momentum in recent years. One such example is the work by Zhang et al. [81], which was aimed at achieving complete tumor ablation via IT injection of HDI-PF127 nanocomposite hydrogel incorporating PTX-loaded chitosan micelles and PEGylated gold nanorods. Qin et al. [82] chose PF127 as the hydrogel matrix and black phosphorus nanosheets as photothermal agents because of their broad absorption in the NIR region and extinction coefficient larger than other 2D materials. While investigating the in vitro release profile of gemcitabine, it was observed that black phosphorus nanosheets accelerated drug release from PF127 hydrogel under NIR irradiation (808 nm, 2.0 W/cm^2^, 10 min). Compared to chemotherapy alone, this hydrogel exhibited a superior antitumor effect and good photothermal effect in BALB/c mice bearing 4T1 xenograft tumors [82]. In another paper [83], the application of NIR light induced on-demand release for up to 14 days after a single administration of PF127 hydrogel with liposomes incorporating DOX and gold-manganese oxide nanoparticles.

In addition to the analyses described in Section 3, the photothermal properties of these poloxamer hydrogels are usually assessed in terms of photo–heat conversion ability and photothermal stability under repeated 808 nm laser irradiation.

Tumor destruction by conventional PDT relies on the photochemical reaction between a light-activated photosensitizer and molecular oxygen to produce ROS, resulting in cell death [84]. However, PDT often fails to completely eradicate tumors due to the limited penetration of currently available photosensitizers into the tissue. Building upon the use of two-photon excitation to improve light penetration, Luo et al. [85] proposed the co-encapsulation of a two-photon absorption compound (T1) and a photosensitizer (pyropheophorbide a) into polymeric micelles combined with PF127. In 4T1 xenograft mice, the obtained hydrogel was shown to inhibit tumor growth in more than 50% (under 1 cm-thick muscle tissue) after IT administration and NIR irradiation. By capitalizing on the synergistic effects of chemotherapy and PDT, Li et al. [86] employed DTX-loaded micelles and black phosphorus nanosheets as a hydrophobic model drug and photosensitizer, respectively, incorporating them into a PF127/PF68 hydrogel. The photodynamic performance of these hydrogels is evaluated by singlet oxygen detection.

### 4.3. Potential Applications in Cancer Immunotherapy

The most recent revolutionary wave in cancer therapy came with immunotherapy and its improvements for patients in terms of survival and quality of life [87]. However, two of the most used classes of immunotherapeutics, cytokines and checkpoint inhibitors, face similar and appreciable delivery challenges. For example, the use of Toll-like receptor (TLR) 7/8 agonists is often limited to IT administration because IV administration can lead to systemic toxicity by stimulating the entire immune system [88]. Therefore, local delivery of TLR 7/8 agonists, such as MEDI9197 [89] and imiquimod [90], is preferred, which can be attained by mixing them with P407 aqueous solutions. Fakhari et al. [89] demonstrated significant antitumor activity of P407 thermogel with MEDI9197 after two IT injections in a B16-OVA melanoma tumor model. In another work [90], imiquimod was first encapsulated in 1,2-dipalmitoyl-sn-glycero-3-phosphatidylcholine liposomes before being incorporated into PF127 hydrogel, with the final delivery system producing promising results in a breast cancer model.

Cytotoxic T-lymphocyte-associated protein 4 (CTLA-4), belonging to the class of checkpoint inhibitors, can also be explored to generate antitumor immune responses. To control the release of anti-CTLA-4 antibodies, Chung et al. [48] pioneered the optimization of CTLA-4 therapy using P407-based injectable hydrogels. The authors observed a significant reduction in serum anti-CTLA-4 levels and effective tumor growth inhibition in CT26 tumor-bearing mice receiving the hydrogel peritumorally. A major feature of the tumor microenvironment is extracellular acidosis, which seems to antagonize the efficacy of immune checkpoint inhibitors. One prominent strategy to alleviate extracellular tumor acidity capitalizes on sodium bicarbonate therapy, but can cause metabolic alkalosis. As such, Jin et al. [91] employed NaHCO_3_-loaded PF127 hydrogel for precise delivery into the tumor, rendering its microenvironment immunologically favorable. Indeed, tumor clearance was improved when treating MC-38-bearing mice with a low dose of immune checkpoint inhibitors after local tumor neutralization with the gel. Very recently, the overall goal of maximizing the therapeutic index of vemurafenib and antagonistic programmed cell death protein 1 antibody used in combination to treat BRAF-mutated melanoma was achieved with PF127-g-gelatin hydrogel and IT administration [92].

Although dendritic cells were initially recognized for their role in antiviral immunity, recent attention has been directed toward their potential to boost the patients’ immune system in the fight against cancer [93]. Still, considering their short viability and low in vivo migration capacity, treatment with adjuvants for the recruitment and maturation of dendritic cells is of great interest. To illustrate, Lemdani et al. [94] designed a mucoadhesive hydrogel consisting of P407 and xanthan gum for IT co-delivery of granulocyte-macrophage colony-stimulating factor and heat-killed *Mycobacterium tuberculosis* to refine the local antitumor immune response. Though administering a solution of these immunomodulatory agents elicited minimal therapeutic effects, their IT injection in the gel led to the infiltration of T cells in the tumor, as well as growth inhibition.

### 4.4. Potential Applications in Cancer Gene Therapy

To date, the utilization of poloxamer hydrogels in local cancer gene therapy is scarce, with only two works being reported.

After coupling conjugated linoleic acid (CLA) with P407, Guo et al. [95] demonstrated that CLA-coupled poloxamer hydrogel could be a local delivery system for PTX with enhanced antitumor efficacy. The evidence of apoptotic cell death inspired these authors to use the obtained hydrogel for combination therapy with PTX and Akt1 shRNA [96]. Knowing that the phosphoinositide 3-kinase/Akt1 signaling pathway has emerged as a target for breast cancer therapy, it is no surprise that the inhibition of Akt1 warrants special attention. In addition to synergistic inhibitory effects in vitro (MDA-MB-231 and MCF-7 cells) and in vivo (MDA-MB-231 xenograft), local treatment with PTX and Akt1 via CLA-coupled PF127 hydrogel was confirmed to decrease Akt1 phosphorylation levels and inhibit angiogenesis [96].

Another promising target for breast cancer is survivin, whose inhibition merits in situ injection to ensure tissue and cell specificity. Taking advantage of electrostatic interactions between a cationic polymer (poly[(R)-3-hydroxybutyrate]-b-poly(2-dimethylamino) ethyl methacrylate) and negatively charged survivin antisense oligonucleotide, Zhao et al. [97] developed a gene delivery nanocomplex subsequently incorporated into injectable PF127 hydrogels for local retention. A single injection was enough to achieve a sustained gene release for up to 16 days and counteract PTX-induced multidrug resistance by silencing up-regulated survivin.

At the end of this subsection, Table 1 summarizes the described injectable poloxamer hydrogels published for the period 2018–2023.

## 5. Conclusions

A myriad of in situ gelling systems triggered by temperature changes has been developed for the IT or PT administration of different therapeutic agents. Among thermoresponsive polymers, poloxamer-based hydrogels are in the spotlight due to their low cost, simplicity of preparation, and compatibility with biological systems [15,98].

Over the past two decades, poloxamer dispersions have been proposed as injectable formulations to assist local cancer treatment using chemotherapy, phototherapy, immunotherapy, and gene therapy. However, the application of injectable poloxamer hydrogels for local tumor administration remains in the proof-of-concept stage, despite promising preclinical (in vitro and in vivo) outcomes. First, it is highly recommended that poloxamers are modified or used in novel combinations of polymers to reduce the erosion rate of conventional poloxamer hydrogels and ensure precise delivery. Moreover, the developed hydrogels are more likely to reach the clinical testing phase if researchers evaluate therapeutic efficacy in larger animals (e.g., monkeys, pigs, and dogs) instead of using rodent models [75]. In addition to clinical translation, the scale-up process from laboratory to industry is also very effortful, the first step being a thorough characterization of the hydrogels including insights into the morphology and thermal properties, and not only rheological and biological analyses [99].

In the future, injectable poloxamer hydrogels are expected to remain an exciting research topic not only for drug delivery but also for tissue engineering [100] and cartilage repair [101,102]. Regarding local cancer therapy, one can envision phototherapy and immunotherapy succeeding chemotherapy as the most applied therapeutic modalities, with increasingly frequent reports. Concurrently, more researchers will follow the trend of combination therapy as a new direction for cancer treatment and establish injectable poloxamer-based hydrogel as a key element in upcoming therapeutic strategies [103].

## Figures and Tables

**Table 1:** Injectable poloxamer hydrogels for intratumoral or peritumoral administration.

Cancer Therapy	Injection Type	Hydrogel Composition	Cancer Cell (In Vitro)	Cancer (In Vivo)	Ref.
Chemotherapy	IT	P407, Topotecan	-	Retinoblastoma (Y79)	[54]
Chemotherapy	-	P407, DOX	MC-38 (colon)	-	[55]
Chemotherapy	IT	P407, Salinomycin	U251	GBM	[47]
Chemotherapy	IT	P407, CDDP NP, LP microspheres	-	Melanoma (B16)	[57]
Chemotherapy	IT	P407, P188, SN-38 liposomes	-	HCC (H22)	[66]
Chemotherapy	IT	P407, P188, DTX micelles	-	Colon (HT-29)	[30]
Chemotherapy	IT	P407, P188, HPMC, NCTD	-	HCC (H22)	[69]
Chemotherapy	IT	P407, DOX, NCTD NP	HepG2	HCC (H22)	[29]
Chemotherapy	IT	P407, HA, HPMC K_4_M, DOX, PTX micelles	-	-	[72]
Chemotherapy	IT	P407, P188, Alginate, 5-FU	-	Colon (CT26-luc)	[41]
Chemotherapy	IT	P407, P188, Xanthan gum, PTX NP	MCF-7	Breast	[38]
Chemotherapy	PT	Heparin-P407, DOX laponite NP	S180	Sarcoma	[73]
PTT	PT	P407, CuS nanodots	4T1	Breast	[77]
PTT	PT	P407, Prussian blue nanospheres	4T1	Breast	[78]
PTT	IT	P407, CA-AuAg NP	4T1 B16F10	Breast Melanoma	[79]
PTT	IT	P407, Ti_3_C_2_ NP	4T1	Breast	[80]
PTT + Chemotherapy	IT	P407, BP nanosheets, Gemcitabine	-	Breast (4T1)	[82]
PDT	IT	P407, T1 and PPa co-encapsulated micelles	4T1	Breast	[85]
PDT + Chemotherapy	IT	P407, P188, BP nanosheets, DTX micelles	-	Breast (4T1)	[86]
Immunotherapy	IT	P407, Imiquimod liposomes	4T1	Breast	[90]
Immunotherapy	PT	P407, CTLA-4 Ab	MC-38 (colon)	Colon (CT26)	[48]
Immunotherapy	IT	P407, NaHCO_3_	-	Colon (MC-38)	[91]
Immunotherapy	IT	P407-g-gelatin, Vemurafenib, PD-1 mAb	D4M B16F10	Melanoma	[92]
Immunotherapy	IT	P407, Xanthan gum, GM-CSF, HKMT	B16 CT26 3LL (Lewis lung carcinoma)	Colon (CT26)	[94]
Gene therapy	IT	P407, Sur-ASON, PHB-b-PDMAEMA	MCF-7/PDR	Breast	[97]

5-FU: 5-fluorouracil; Ab: antibody; BP: black phosphorus; CA-AuAg: iota carrageenan-capped gold-silver; CDDP: cisplatin; CTLA-4: cytotoxic T-lymphocyte-associated protein 4; DOX: doxorubicin; DTX: docetaxel; GBM: glioblastoma multiforme; GM-CSF: granulocyte-macrophage colony stimulating factor; HA: hyaluronic acid; HCC: hepatocellular carcinoma; HKMT: heat-killed Mycobacterium tuberculosis; HPMC: hydroxypropyl methylcellulose; IT: intratumoral; LP: losartan potassium; NCTD: norcantharidin; NP: nanoparticles; P188: poloxamer 188; P407: poloxamer 407; PD-1 mAb: programmed cell death protein 1 monoclonal antibody; PDT: photodynamic therapy; PHB-b-PDMAEMA: poly[(R)-3-hydroxybutyrate]-b-poly(2-dimethylamino) ethyl methacrylate; PPa: pyropheophorbide a; PT: peritumoral; PTT: photothermal therapy; PTX: paclitaxel; SN-38: 7-ethyl-10-hydroxycamptothecin; Sur-ASON: survivin antisense oligonucleotide.

## Data Availability

Not applicable.
